# Polyvinyl alcohol/silver electrospun nanofibers: Biocidal filter media capturing virus‐size particles

**DOI:** 10.1002/app.51380

**Published:** 2021-07-16

**Authors:** Magda Blosi, Anna Luisa Costa, Simona Ortelli, Franco Belosi, Fabrizio Ravegnani, Alessio Varesano, Cinzia Tonetti, Ilaria Zanoni, Claudia Vineis

**Affiliations:** ^1^ National Research Council of Italy Institute of Science and Technology for Ceramics (CNR‐ISTEC) Faenza Italy; ^2^ National Research Council of Italy Institute of Atmospheric Sciences and Climate (CNR‐ISAC) Bologna Italy; ^3^ National Research Council of Italy Institute of Intelligent Industrial Technologies and Systems for Advanced Manufacturing (CNR‐STIIMA) Biella Italy

**Keywords:** electrospinning, fibers, filter technology, nanocrystals, nanoparticles, nanowires, separation techniques

## Abstract

In response to the nowadays battle against SARS‐CoV‐2, we designed a new class of high performant filter media suitable to advance the facemask technology and provide new efficient widespread solutions against virus propagation. By means of the electrospinning technology we developed filter media based on polyvinyl alcohol (PVA) nanofibers doped with AgNPs combining three main performance requirements: high air filtration efficiency to capture nanometer‐size particles, low airflow resistance essential to ensure breathability and antimicrobial activity to inactivate aerosolized microorganisms. PVA/AgNPs electrospun nanofibers were produced by electrospinning the dispersion of colloidal silver into the PVA water solution. A widespread physicochemical characterization was addressed to the Ag colloidal suspension. The key functional performances of the electrospun nanofibers were proven by water stability, antibacterial activity, and filtration efficiency and pressure drop measurements performed under conditions representative of facemasks. We assessed a total bacterial depletion associated to a filtering efficiency towards nano‐aerosolized particles of 97.7% higher than required by the EN149 standard and a pressure drop in line with FFP1 and FFP2 masks, even at the highest filtration velocity. Such results pave the way to the application of PVA/AgNPs electrospun nanofibers in facemasks as advanced filtering media for protecting against airborne microorganisms.

## Graphical abstract

The battle against SARS‐CoV‐2 requires affordable solutions to block virus propagation before infecting cells. Electrospinning is one of the promising technologies, providing patches (filter media) able to capture nanometer‐size particles and inactivate aerosolized microorganisms, if doped with antimicrobial ingredients, like Ag nanoparticles. In this work we produced PVA/Ag electrospun nanofibers and found high filtering efficiency, low air drop‐pressure and excellent antibacterial properties, making them optimum candidates to be incorporated in personal protective equipment face masks.

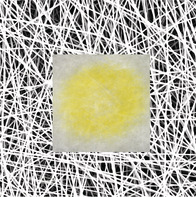

## 1 INTRODUCTION

Materials composed with sub‐micron or nanometer‐sized polymer fibers are characterized by interesting properties such as large surface‐to‐volume ratios, small pore sizes and superior surface properties. These outstanding characteristics mean that layers of polymer nanofibers (NFs) are excellent candidates for applications such as high efficiency filters, breathable protective clothing and facemasks, designed to protect from airborne biohazards and particulate matters.^1^

In a situation of worldwide public‐health emergency, in the absence of any known efficient therapy and access to vaccine, to control the dramatic consequence of virus SARS‐CoV‐2 infection (Covid‐19), the production of new protective devices such as masks is urgent. The functionalization of filter media with antibacterial and virucidal agents, as silver nanoparticles (Ag NPs), could represent a success in preventing or even suppressing the spread of the virus, thanks to their incorporation in the medical personal protective equipment like as facemasks.^1^, ^2^ Electrospinning is one of the most promising processing technologies to embed NPs in a wide range of polymer NFs.

The typical basic electrospinning setup comprises an electrically charged tip, a collector (generally grounded) and a solution feed.^2^ Electrospinning process can be further developed for mass production of continuous nanofibers of various polymers and composites materials.^3^, ^4^, ^5^, ^6^, ^7^

The diameter of electrospun NFs depends on process parameters such as solution properties (viscosity, conductivity and surface tension), ambient conditions (temperature and humidity) and process conditions (solution flow rate, electric voltage, tip‐to‐collector distance, tip size).^2^, ^8^, ^9^, ^10^

Electrospun NFs have been proposed as a promising component of filter media for high filtration efficiency.^11^, ^12^ Traditional filter media may have difficulties to simultaneously achieve high efficiency and low pressure drop (or high air permeability). On the contrary, a layer of electrospun nanofibers, having small thickness and a large surface area, enable to significantly improve the filtration efficiency without increasing the pressure drop,^13^ so matching the face masks technical requirements.

The mechanical filtration theory for fibrous filters is based on the single fiber collection efficiency given by diffusion, inertial impaction, interception, and gravitational settling (which can be neglected in these applications).^14^ Impaction is negligible for small particles (below 0.2 μm), where diffusion is the only important mechanism, but increases rapidly for particles larger than 0.5 μm. In filtration theories, it is commonly assumed that the individual filtration mechanisms discussed above are additive and independent of each other. Therefore, the overall single‐fiber collection efficiency can be written as the sum of individual single‐fiber efficiencies contributed by the different mechanisms.^14^ The smaller the single fiber diameter the higher is the air filtration efficiency.

In order to introduce electrospun nanomaterials in filtering devices, NFs were deposited on flat porous substrates, such as fabrics or non‐wovens, to combine advantages of both materials.^4^, ^13^, ^15^ Heikkilä et al^16^ reported that a polyamide‐66 nanofibers layer with density of 0.50 g m^−2^ removed more than 90% of airborne particles with sizes from 200 nm to 10 μm, regardless of textile substrates. On the contrary, the substrates influenced the pressure drop. With a layer of 0.50 g m^−2^ of polyamide‐66 nanofibers, the lowering of pressure drop (124 Pa) was measured on a 50 g m^−2^ cellulose fiber non‐woven. Zhang et al^17^ investigated the influence of several electrospinning parameters (i.e., tip‐to‐collector distance, solution concentration, flow rate) on filtration efficiency (in capturing sub‐micron particles) and pressure drop. Polyamide‐6 electrospun NFs were compared with two meltblown filter media with surface density of 68 and 81 g m^−2^. Electrospun NFs with a surface density of ~16.5 g m^−2^ resulted to be more efficient in capturing small particles with higher pressure drop than conventional non‐wovens. Another work on electrospun polyamide‐6 nanofibers^18^ showed a filtration efficiency close to 90% in removal of 0.5 μm‐sized particles and higher efficiencies (close to 100%) for larger particles (1, 6, and 10 μm). The highest filtration efficiency (on 75 nm count median particles size) and lowest pressure drop (at 85 L/min airflow velocity) obtained with polyacrylonitrile NFs produced by a needleless electrospinning apparatus were 94% and 140 Pa, respectively.^3^ High collection efficiencies of nanoparticles (from 7.37 to 150 nm) were recorded on polyethylene terephthalate electrospun fibers with average diameter from 1.29 to 0.67 μm.^4^ The addition of SiO_2_ nanoparticles in polyethylene terephthalate solution before electrospinning reduced the average sizes and increased pressure drops.^5^

Nevertheless, despite to filter efficiency, the pressure drop across the filter media represents a key parameter to be considered. Ideally, filters that exhibit a high filtration efficiency at a low pressure drop are the most desirable ones. Both aspects are considered into the Quality Factor (QF), which is often used to evaluate the filtration performance of filters,^14^ as defined as Equation 1:

(1) $$\mathrm{QF}=\frac{-\ln\;\left(1-E\right)}{\mathrm{\Delta P}}$$

where ∆P is the pressure drop (in Pa) and E is the filtration efficiency.

The state‐of‐the‐art for filtration processes deals with higher capture efficiency of particles, without increasing the pressure drop. Filtration efficiencies and pressure drops increased as the NFs surface density increased. Recently, a study ^6^reported that the introduction of large particles inside the NF layers as spacers reduced both filtration efficiency and pressure drop, but the magnitude of the reduction in the pressure drop was higher than efficiency, so filters had better QFs. The filtration efficiency reached almost 100% when 2.4 g m^−2^ of NFs were collected, but the pressure drop risen up to 1530 Pa on meltblown non‐woven. Polyvinyl alcohol (PVA), among other polymers, is electrospinnable from water solutions (no harmful or toxic solvents) in a wide range of concentrations and ambient conditions. Despite its solubility in water, PVA NFs are stable in wet conditions below 80°C. Moreover, PVA NFs can be crosslinked to further increase the environmental stability.^19^, ^20^

In addition, polymer NFs have been proposed as supports for chemical (e.g. photodegradation)^21^, ^22^ and biological (e.g., antibacterial activity)^23^, ^24^, ^25^ active phases to obtain enhanced properties on porous materials, including high‐efficiency filter media. In particular, antibacterial NFs are easily produced incorporating biocides in electrospinning solutions. Examples range from antibacterial biological complexes,^26^ antibacterial polymers (such as chitosan),^27^ or nanoparticles^28^, ^29^ (NPs).

Silver is one of the most studied components for producing antibacterial polymer composite nanofibers. Silver nanoparticles (Ag NPs) are known for their antimicrobial properties, and their use in commercial products is increasing. In fact, they are already widely found as antiseptic additives in packaging, fabric, and are also ideal candidates as additives for tile coatings.^30^ Ag NPs are thought to exert their antimicrobial effect through the gradual release of Ag^+^ ions and being less susceptible to sequestration by chloride, phosphate, proteins and other cellular components than silver ions alone are proven to be more effective.^31^

Recent studies have shown the improvement of anti‐microbial performance of face mask using silver based nanoparticles.^7^, ^8^, ^9^, ^10^ The enhancement of antibacterial property of commercially available masks by treating it with Ag nanoparticles was demonstrated by Hiragond and collegues. Different concentration of AgNPs were applied by simple soaking on a surgical mask. The best results were obtained for face masks treated with 100 ppm Ag NPs that showed enhanced antibacterial activity of commercial face masks. They demonstrated the reduction of the contamination of face masks giving a longer duration of wearability through a simple coating method.^11^ Similarly, a novel, easily processed and cheap coating for a nylon fabric with antimicrobial characteristics was developed and proposed by Botelho and colleagues. The fabric was impregnated with chitosan and silver nanoparticles by simply dipping into a mixture of both components. The impregnated fabric possessed bactericidal activity higher for Gram‐positive *Staphylococcus aureus* than for Gram‐negative *Pseudomonas aeruginosa* bacteria. Poor washing fastness makes the novel fabric suitable in single‐use face masks.^12^ More recently, nano‐developed disinfectant was designed by Valdez‐Salas et al to uniformly coat hydrophobic surgical masks with AgNPs, promoting antimicrobial activity, as well as viral inactivation efficacy for healthcare protection. The proposed strategy allows the incorporating AgNPs to medical grade textiles fibers of the surgical masks protecting against microbial penetration and adhesion.^13^

Moreover, Ag NPs represent a very good candidate to be integrated in electrospun NFs thanks to its small size and very large surface area that improves the surface specific reactivity. Some examples of Ag NPs successfully embedded in electrospun PVA NFs, in different ways, are reported in literature.^20^, ^32^, ^33^

In the present paper we developed PVA/Ag electrospun NFs aiming to achieve a new class of filtering media for facemask intended use. We applied a water‐based Ag NPs suspension prepared by an eco‐friendly and easily scalable process^34^ and providing Ag NPs embedded into hydroxyethyl cellulose (AgHEC). PVA/AgHEC solution was electrospun in NFs on spunbonded polypropylene non‐wovens. We achieved filtering media characterized by outstanding performance requirements for facemask purposes as like as high air filtration efficiency and low pressure drop as expressed by QF values comparable with or better than commercial filter media. Such key technical goals, further implemented by the excellent antimicrobial activity ensured by AgHEC NPs, paved the way for future promising application perspectives.

## 2 EXPERIMENTAL

### 2.1 Filter production

Commercial PVA with a weight‐average molecular weight of 130,000 g mol^−1^ was purchased from Sigma‐Aldrich (Italy). PVA powder was dissolved as received in water at 90°C under mild stirring until the solution appeared transparent (about 2 h). Then PVA solution was slightly cooled down at ambient temperature (about 20°C) under stirring. The concentration was 15 wt%/vol%.

Silver nanoparticles were synthesized at room temperature reducing a solution of AgNO_3_ by means of hydroxyethyl cellulose (HEC) which acts as capping agent as well. The reduction synthesis was catalyzed by NaOH according to the patented procedure,^34^ producing AgHEC. The AgHEC NPs are characterized by small spherical particles (hydrodynamic diameter around 60 nm and positive surface charge, zeta potential ≈ + 4.5 mV).^35^

Colloidal AgHEC NPs dispersion (0.1 wt%) was added to the PVA solution at a volume ratio 1:1. The final hybrid PVA/AgHEC solutions were kept under stirring for at least 2 h in order to ensure complete mixing before electrospinning.

PVA/AgHEC solutions were electrospun using an electrospinning equipment consisting of a high‐voltage generator (Spellman SL300P) electrically connected to a 27G metal tip (Butterfly infusion set by Hospira, UK), a metering pump (KDS 200 from KD Scientific) feeding the solution to the metal tip (0.4 mm internal diameter) and a flat metal collector (500 mm by 500 mm) electrically connected to a second high‐voltage generator (Spellman SL300N). A 23‐gsm polypropylene spunbonded non‐woven with an average fiber size of 16 ± 4 μm (supplied by Soft NW, Italy) were cut in squares with the same size of the collector and stuck on it as a substrate suitable for handling the nanofiber layers.

The addition of AgHEC nanoparticles seems to destabilize the fiber process formation probably due to the interaction of the components, namely HEC and PVA. Multiple jets were ejected from the metal tip instead of a single jet as usual. A simple grounded collector was not able to gather all the fibers produced. Therefore, the collector was electrically charged as used in multi‐jet electrospinning.^6^

The PVA/AgHEC solutions were processed at voltages of +30 kV at the tip and − 5 kV at the collector with a working distance from the tip to the collector of 20 cm and a flow rate of 0.02 ml/min. The ambient conditions were 22 ± 2°C temperature and 35 ± 5% relative humidity. Electrospun nanofibers were collected on the non‐woven. Each deposition lasted 1 h.

At these conditions, the electrospinning was able to cover an average surface of 448 ± 33 cm^2^. Such a large deposition area is due to the multitude of jetting produced during the process. The jets were not stable in position at the tip and each of them resulting single deposition area overlapped each other producing a large circular surface coated with yellow NFs. The NF layer density was calculated to be 2.0 g m^−2^. The calculated nanofiber densities were confirmed by mass measurements as average of six measures to be 1.83 ± 0.34 g m^−2^. PVA/AgHEC NF‐coated samples were treated in an oven at 155°C for 3 minutes to fix the nanofibrous porous structure.^20^

### 2.2 Physicochemical characterization

The hybrid PVA/AgHEC solution was characterized by means of Zetasizer nano ZSP (model ZEN5600, Malvern Instruments, UK), after appropriate dilution 1:5 in water. The particle size distribution was determined by dynamic light scattering (DLS) technique. Zeta potential measurements were performed by electrophoretic light scattering (ELS). The Smoluchowski equation was applied to convert the electrophoretic mobility to zeta potential. DLS analysis provides also a polydispersion index parameter (PDI), ranging from 0 to 1, quantifying the colloidal dispersion degree; for PDI below 0.2 a sol can be considered monodispersed. The hydrodynamic diameter and zeta potential values were obtained by averaging three measurements.

Morphological analysis was performed by transmission electron microscopy (TEM). One drop of AgHEC NPs diluted in water was deposited on a carbon film‐coated copper grid and then air dried. The sample was examined by FEI TECNAI F20 microscope operating at 200 keV.

Diffraction pattern was collected on the synthesized AgNPs dripped on a glass slide and dried at 100°C for 15 min. Analyses were performed by the Bruker D8 Advance diffractometer (Germany) operating in θ/2θ configuration, with a LynxEye detector (10–80° 2θ range, 0.02 stepsize, 0.5 s time‐per‐step equivalent). Fourier‐transform infrared ‐ Attenuated total reflection (FTIR‐ATR) spectroscopy was carried out using Nicolet iS5 spectrometer (Thermo Fisher Scientific Inc., Waltham, MA), with a resolution of 1 cm^−1^ by accumulation of 24 scans, covering the 4000 to 400 cm^−1^ range and using a diamond ATR accessory model iD7.

PVA/AgHEC electrospun fibers microstructure was observed by scanning electronic microscopy analysis using a Carl Zeiss Sigma (Germany) field emission scanning electron microscope (FESEM) on the flesh electrospun NF‐coated filters and a Carl Zeiss Sigma (Germany) EVO 10 Scanning Electron Microscope (SEM) on the NF‐coated filters after the contact with water. Filter patches were pasted on the aluminum stub and sputtered with a 2 nm gold layer to ensure conductivity. The average fiber diameter was calculated by means of an image analysis program (ImageJ by NIH) and averaged on more than 300 measures.

The total silver amount embedded into the electrospun PVA/AgHEC NF‐coated filters was assessed by an Agilent Technologies, Inductively Coupled Plasma Optical Emission Spectroscopy ICP‐OES 5100 equipped with vertical dual view apparatus after mineralization. For mineralization, patches of about 0.5 g of PVA/AgHEC NF‐coated filter were microwave treated for 20 min at power ranging from 250 to 600 W in presence of 6 ml of 65% nitric acid (Merck) with 1 ml of 30% hydrogen peroxide (Merck). After microwave digestion, the samples were filtered by 0.22 μm cellulose membrane in order to remove coarser residual fibers. Then milliQ water was added reaching the final volume of 15 ml. The so‐prepared samples were analyzed by ICP‐OES. A calibration curve obtained by dilution of silver standard solution (Sigma Aldrich, Italy) for ICP‐OES in the range 0–100 mg L^−1^. The limit of detection (LOD) for silver at the operative wavelength of 328.068 nm was 0.01 mg L^−1^.

### 2.3 Water stability test

To assess water stability of the PVA/AgHEC NFs, both as‐spun NFs and thermal‐treated samples were put in contact with water at 25–27°C for 24 h. About 1 cm^2^ of each sample was stuck on the bottom of a Petri dish (3.5 cm diameter) and 5 ml of demineralized water were add. After the test, the samples were dried at room temperature before SEM observations, as previously described.

### 2.4 Antibacterial tests

Biocidal action of electrospun PVA/AgHEC NFs was assessed following ASTM E2149 procedure against Gram‐negative bacteria *Escherichia coli* ATCC 11229 and Gram‐positive bacteria *Staphylococcus aureus* ATCC 6538. The test culture was incubated in a nutrient broth for 24 h, and then it was diluted to a concentration of 1.5–3.0 × 10^5^ CFU ml^−1^ (working solution). About 0.5 g of electrospun PVA/AgHEC NFs were transferred to flask containing 25 ml of the working solution. This condition corresponds to spread 91 ml of a liquid on one square meter of filter containing nanofibers. All flasks were shaken for 1 h at 190 rpm. After a series of dilutions, 1 ml of the solution was plated in nutrient agar. The inoculated plates were incubated at 37°C for 24 h and surviving cells were counted. The biocidal action was expressed in percent bacteria reduction by counting the surviving cells after contact with the test specimen (A) compared to the number of bacterial cells in the working solution (B), according to the Equation 2:

(2) $$\text{Reduction}\;\left(\%\right)=\left[\;\frac{\left(B-A\right)}{B}\right]x\;100$$

### 2.5 Filtration tests

Three different sheets of NFs were considered and tested, for filtration, in triplicate. From each sheet, a five‐layer stack is obtained by cutting five discs of 5 cm in diameter and positioned one upon each other (all the results are expressed as an average of 5‐layer samples). The experimental filtration velocities were 5.5 and 16.7 cm s^−1^. The latter was considered as representative of masks with low respirable surfaces.

The air filtration tests of electrospun PVA/AgHEC NFs were assessed by means of different measurement techniques. The filtration efficiency as a function of the particle size was carried out by means of an SMPS system (L‐DMA model 5400 Vienna type, Grimm Aerosol Technik GmbH) (setup 1), which allowed the evaluation of the Most Penetrating Particle Size (MPPS).

The filtration efficiency of 1 μm monodisperse polystyrene latex particles (PSL, Duke Standards, Thermo‐Fisher Scientific Particle Technology) was obtained by means of an Optical Particle Counter (OPC model 11‐A, Grimm Aerosol Technik GmbH) (setup 2). Finally, the filtration efficiency on the total particle mass concentration was assessed by an aerosol photomer (DustTrack, model 8530, TSI) (setup 3). DustTrack can measure aerosol mass concentration in the range 0.1–10 μm according to the data sheet. This technique is similar to the EN149‐2009 standard used in Europe for facemask testing.

**FIGURE 1:**
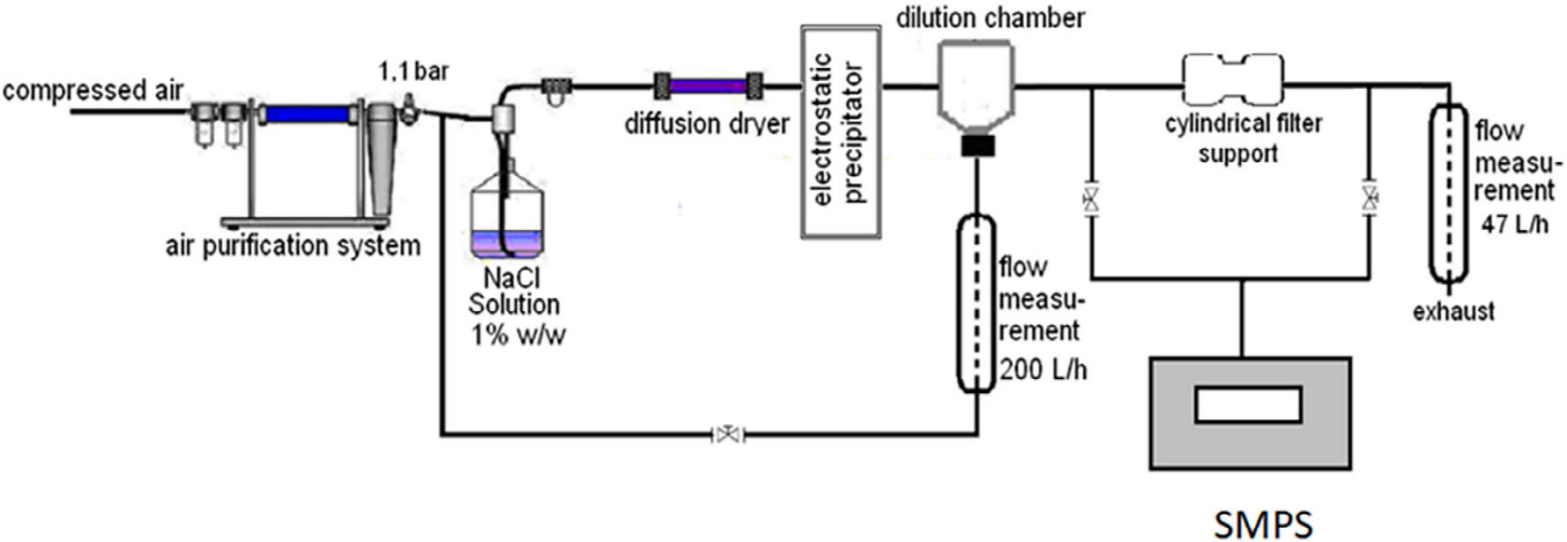
Experimental set up to measure the filtration efficiency of PVA/AgHEC nanofibers. Other configurations were obtained by changing the SMPS analyzer with the OPC or the photometer [Color figure can be viewed at wileyonlinelibrary.com]

NaCl solution at 1% wt/wt was atomized by means of a collision atomizer followed by a diffusion silica gel column to evaporate the water from the droplets. The generated aerosol particles were comparable to a virus size distribution, within the range 10–700 nm with a count median diameter around 70 nm and a mass median diameter of 0.5 μm. The pristine monodisperse PSL particles suspension, once diluted by a factor of 80, was atomized and sent to the test ring. Because of the low particle number concentration obtained with PSL spheres, bends and other obstruction parts of the circuit were removed included the electrometer which was left out from these experiments. Figure 1 shows the experimental scheme of setup 1. In setups 2 and 3, the OPC or the photometer sensor, respectively replaces the SPMS device.

Filtration efficiency values were calculated from three sequential measurements (upstream‐downstream‐upstream), and the considered upstream particle number (or mass) concentration was the average of the two measurements. The filter pressure drop was measured by means of a differential pressure gauge (Testo 512).

## 3 RESULTS AND DISCUSSION

### 3.1 Physicochemical characterization of prepared AgNPs

Ag NPs were prepared according to a patented eco‐friendly and easily scalable process^14^ conducted at room temperature promoting highly stable and concentrated water‐based suspensions, where hydroxyethylcellulose acts both as reducing and chelating agent. AgHEC suspension was easily coupled in solution with PVA to be electrospun.

**FIGURE 2:**
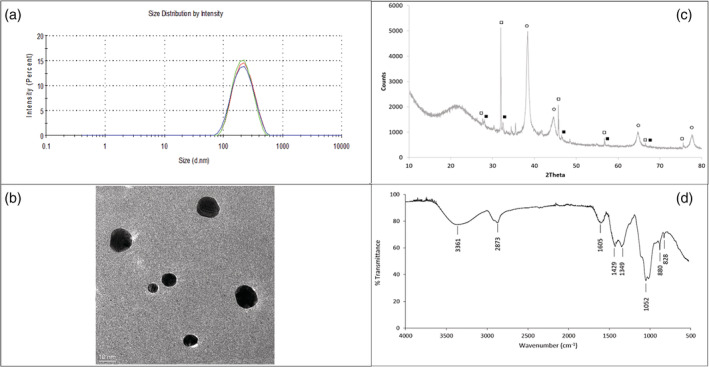
(a) Particle size distribution of hybrid PVA/AgHEC solution; (b) TEM image; (c) XRD diffrattogramm (■ = AgCl; □ = NaCl; ○ = ag) and (d) FTIR‐ATR spectrum of AgHEC [Color figure can be viewed at wileyonlinelibrary.com]

The electrospinnable hybrid PVA/AgHEC solution was colloidally analyzed through DLS (Figure 2(a)) and ELS techniques. The suspension was characterized by a hydrodynamic diameter of 176.3 ± 3.6 nm with PDI equal to 0.2 and a zeta potential of +9.0 ± 0.3 mV. The good correlation between curves of three measurements (Figure 2(a)), the low standard deviation value and low PDI demonstrated the presence of a colloidally stable and monodispersed suspension. The zeta potential value found corresponds to the positive surface charge of Ag NPs surrounded by HEC coating. TEM image (Figure 2(b)) shows well dispersed spherical particles with primary diameter ranges from 3 to 20 nm, lower than DLS hydrodynamic diameter, confirming the presence of HEC coating on AgNPs. Figure 2(c) shows the XRD diffrattogramm of AgHEC NPs. Metallic silver is the predominant phase represented by the typical face‐centered cubic lattice peaks with an equally abundant amount of NaCl, formed as byproduct from Cl^−^ (counter ion of hydroxyethylcellulose compound) and Na^+^ (coming from NaOH, added as catalyst of the AgNO_3_ reduction reaction). Negligible amounts of unreacted AgCl were found as consistent with an almost total reaction yield. FTIR‐ATR analysis shows typical spectrum of HEC compound (Figure 2(d)). A good correspondence with HEC spectrum reported in literature was found.^3^, ^15^, ^16^ As is seen in Figure 2(d), the spectra of HEC are determined at 3361, 2873, 1605, 1429, 1349, 1052, 880, and 828 cm^−1^. The stretching vibrations of O─H and C─H were found at 3361 and 2873 cm^−1^, respectively. The band at 1605 cm^−1^ corresponds to the bending mode of the naturally absorbed water. The spectra showed the absorption bands around 1429 and 1349 cm^−1^ which were attributed respectively to the ─CH_2_ and to the O─H bending. The band at 1052 cm^−1^ is the band of C─O stretching vibrations characteristic of the cellulose skeleton. The bands at 880 and 828 cm^−1^ could be derived from b‐glucosidic linkages between glucose units in cellulose.

### 3.2 Morphology and water stability

**FIGURE 3:**
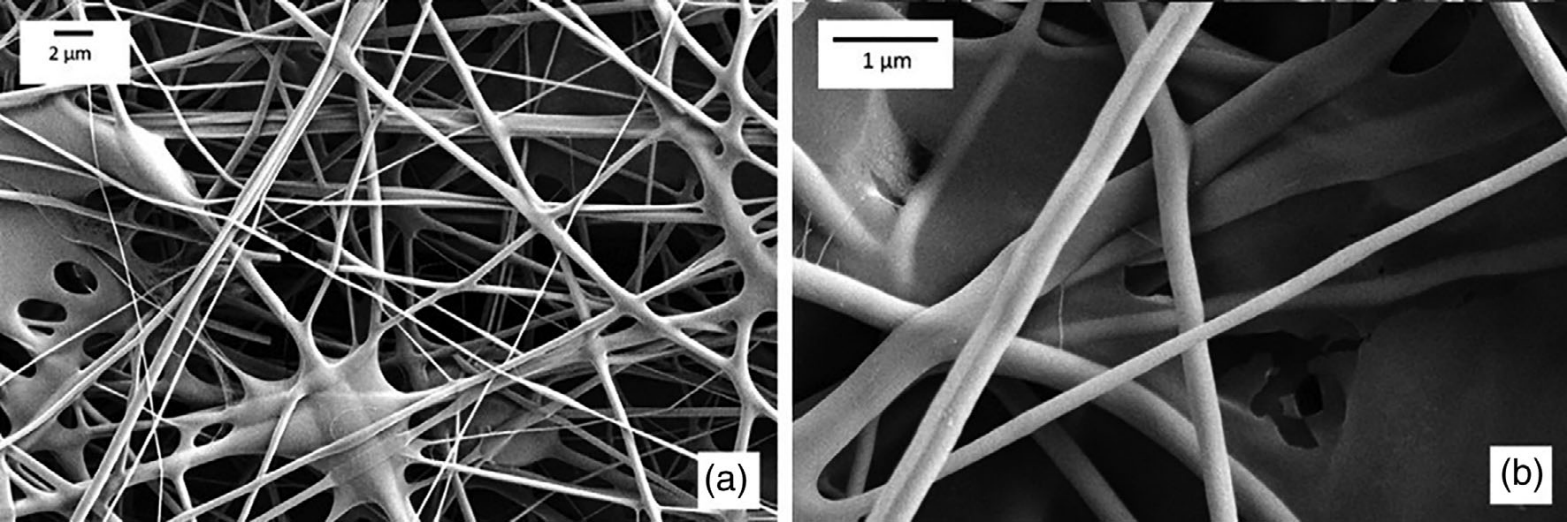
FESEM images of PVA/AgHEC NF‐coated filters at low (a) and high magnification (b)

**FIGURE 4:**
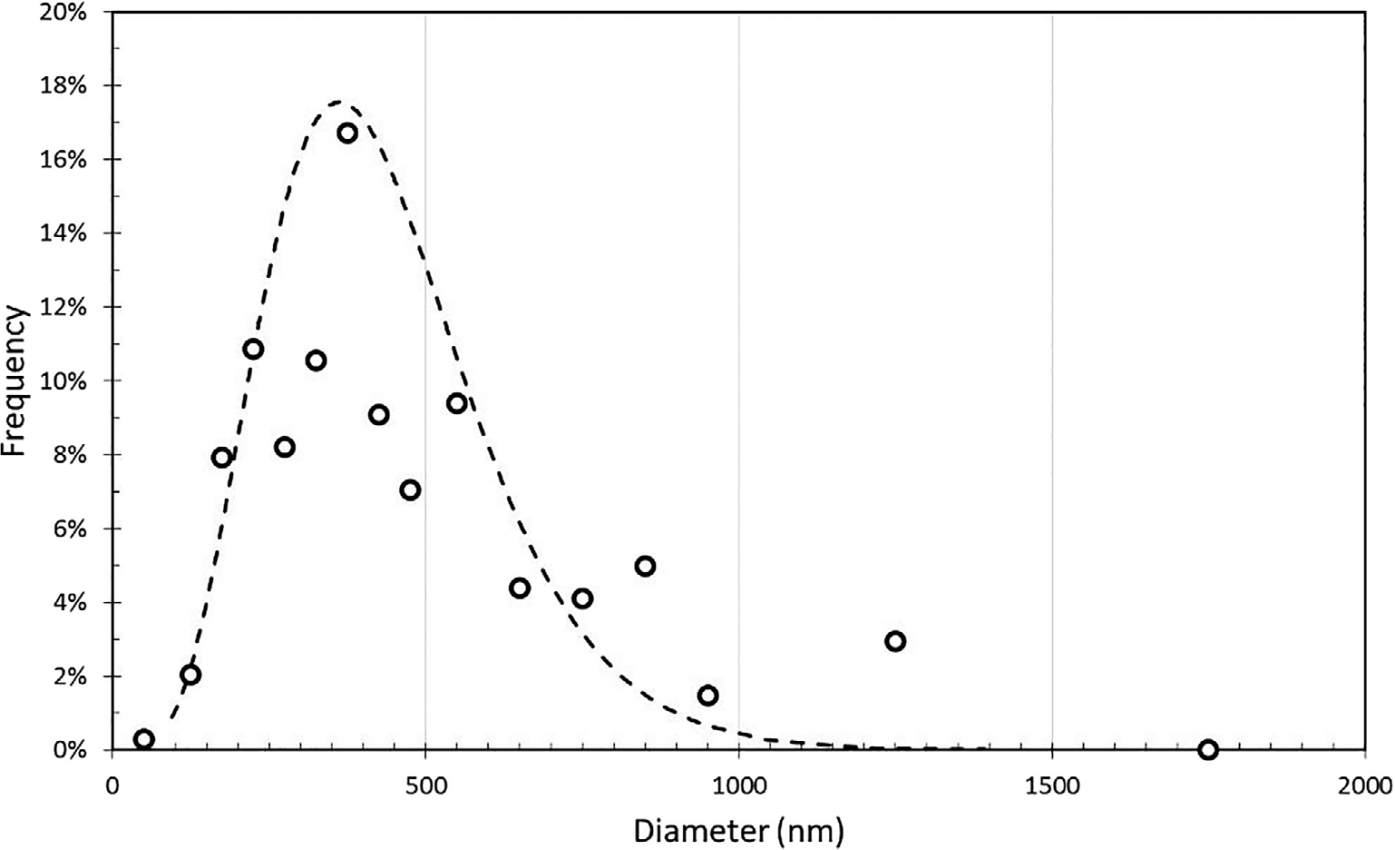
Diameter distribution of PVA/AgHEC NFs

FESEM images in Figure 3 showed the typical porous structure characterized by stretched and interconnected nanofibers with broad diameter distribution ranging from 100 nm to ~1 μm. The widening of the diameter distribution is probably due to the rising of multiple jetting during electrospinning that produces fine fibers. The average fiber diameter is 434 nm. Figure 4 shows the diameter size distributions clustered in 50 nm for the fiber size range 0–500 nm, 100 nm for the range 500–1000 nm and 500 nm for fiber size above 1000 nm. The curves were obtained by fitting the measures with two‐parameter gamma distribution. The pore size of a fiber‐based porous structure is proportional to the fiber diameter itself, so that small pore size achieves high filtration efficiency and conversely decreases the pressure drop of the filter media.^17^ In agreement with a previous paper,^2^ PVA NFs with fiber diameter in the range of several 100 μm have micron‐sized opening pores with an equivalent average pore size close to 1 μm.

**FIGURE 5:**
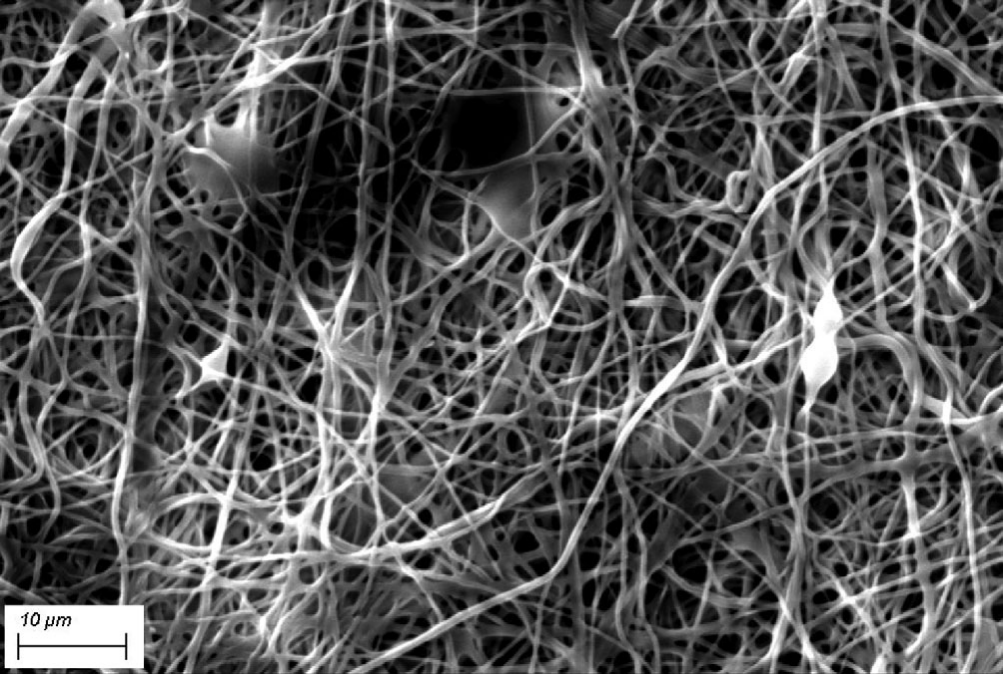
SEM picture of heat‐treated PVA/AgHEC NFs after water contact for 24 h. scale bar: 10 μm

The thermal treatment effect on the PVA/AgHEC NFs stabilization has been evaluated after a contact with water for 24 h. The samples not thermally treated completely loss the porous structure (picture not reported). On the contrary, the NFs subjected to the thermal treatment maintain their shape and porous structure, as shown in SEM image (Figure 5), demonstrating an excellent stability to water.

The amount of silver in PVA/AgHEC NFs was assessed by means of ICP‐OES analysis performed on the electrospun patches after acid digestion and was estimated to be 32.2 ± 18.4 mg m^−2^. If expressed in mass, the experimental concentration of silver in the NF layer tested was ~14 mg g^−1^, in line with the theoretical value of 13.3 mg g^−1^.

### 3.3 Antibacterial activity

The antibacterial test results confirmed that AgHEC NPs integrated into the nanofibers matrix ensures an excellent biocidal action against both Gram‐positive and Gram‐negative bacteria. In fact, nanofibers enriched of silver NPs showed bacteria reduction percentages of 99.6% against *S. aureus* and 100% against *E. coli*.

### 3.4 Filtration tests

**FIGURE 6:**
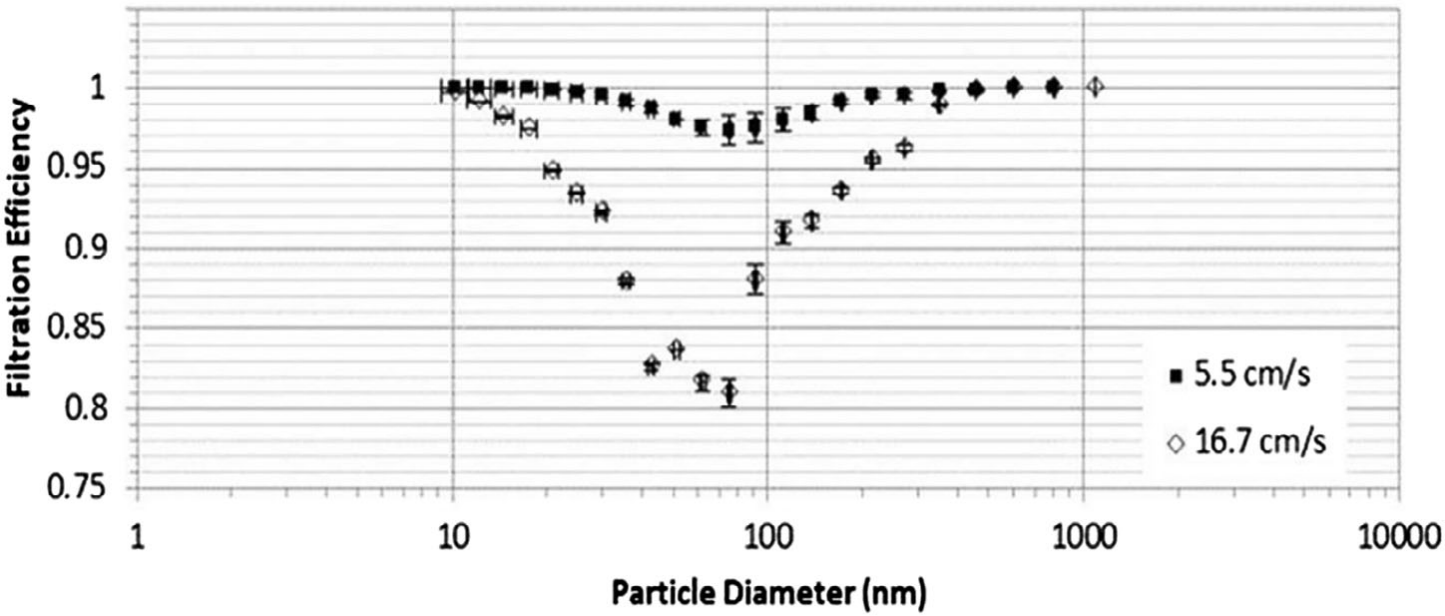
Filtration efficiency as a function of NaCl particles diameters for two different filtration velocities

The filtration efficiency of 5‐layer samples of PVA/AgHEC NF were evaluated as a function of aerosolized particle diameters, for two different filtration velocities of 5.5 and 16.7 cm s^−1^, representative of masks with a low filtration surface. The most particle penetrating size (MPPS) is around 75 nm. As Figure 6 shows, the filtration efficiency is high (>95%) at filtration velocity of 5.5 cm s^−1^ for all the particles size. While, the efficiency decreases for filtration velocity of 16.7 cm s^−1^ with a minimum of about 80%. The averaged pressure drop values measured at 5.5 and 16.7 cm s^−1^ were 59 and 230 Pa, respectively. The corresponding QF values, at the MPPS size, were 0.061 and 0.007 Pa^−1^, higher or comparable with the values reported in literature for electrospun filter media.^11^, ^36^ The QF values for PM2.5 size fraction were 0.067 and 0.017 Pa^−1^, respectively. Gao H. et al, characterized polyacrylonitrile (PAN) three‐dimensional composite membrane fabricated via multi‐jet free surface electrospinning for PM2.5 masks. The obtained QF values at 5.3 cm s^−1^ filtration velocity where about 0.039 for PM2.5 filtration.^18^ Bilayer beaded electrospun nanofiber membranes developed for respiratory air filtration showed lower QF values at comparable filtration velocity (8 cm s^−1^) showed QF values below 0.03 Pa^−1^ (Kadam V., et al).^17^

The comparison between the proposed 5‐layer samples and EN149‐2009 standard requirements for filtration efficiency have been considered. The filtration efficiency and the pressure drop required by the standard are indicated in Table 1 and compared with the performances of 5‐layer samples, at two different filtration velocities. The 5‐layers samples showed pressure drop and filtration efficiency values that are within the range of FFP1 and FFP2 masks, even at the highest filtration velocity of 16.7 cm s^−1^. From these results, it can be inferred that such a filter media could be a good candidate for FFP1 and FFP2 masks. For example, at 16.7 cm s^−1^ the obtained total filtration efficiency was 97.7% higher than 94% as required by the EN149 standard.

**TABLE 1:** Comparison between the 5‐layer samples of PVA/AgHEC NF and the EN149‐2009 requirements for pressure drop (breathability) and filtration efficiency at 95 L/min

EN149‐2009 requirements			Present work		
Mask	Pressure drop (Pa)	Efficiency (%)	Air velocity (cm s^−1^)	Pressure drop (Pa)	Efficiency (%)
FFP1	<210	>80	5.5	59	99.6 ± 0.2
FFP2	<240	>94	16.7	230	97.5 ± 0.2

**FIGURE 7:**
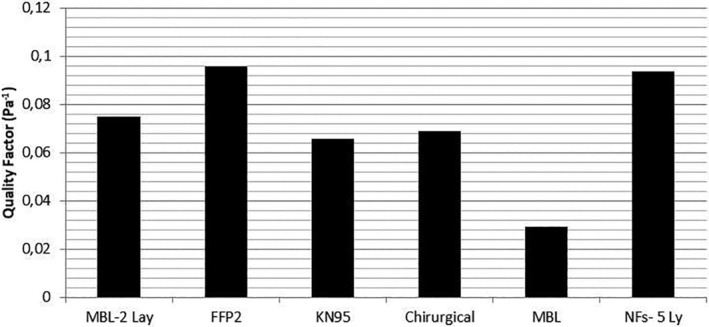
QF values of different materials used for commercial facemasks. Present study: NFs‐5Ly

Figure 7 shows a comparison based on QF values between the 5‐layer samples of PVA/AgHEC NF of the present study with commercial filter media (i.e., single and two layers of meltblown non‐woven, FFP2 and KN95 masks, and a surgical mask). The tests were carried out using the same setup, at the same experimental conditions of 5.5 cm s^−1^ filtration velocity. Results show comparable performance against commercial FFP2 with the advantage that PVA/AgHEC NF have antibaterical activity. Furthermore, their filtration characteristics, being mechanical based, could make these electrospuns NFs an ideal andidate for high efficient potential reusable protective masks. The filter media proposed in this work showed a QF value comparable with or better than commercial filter media.

Finally, we also tested the filter efficiency of 5‐layer samples, towards 1 μm PSL monodisperse aerosolized particles. Resulting the downstream particle number concentration below the OPC detection limit (1 L^−1^) and considered an upstream particle number concentration of about 2 × 10^5^ L^−1^ we could establish a lower limit for the filtration efficiency, at 1 μm, of 99.95%.

## 4 CONCLUSIONS

Silver nanoparticles embedded in ethylcellulose matrix were synthesized by means of a green and easily scalable process and were dispersed in a PVA polymer water solution to be electrospun over spunbonded polypropylene non‐wovens. Samples obtained by stacking five layers of the electrospun sheets provided promising performances, fulfilling typical valuable requirements for facemask applications. We assessed a high air filtration efficiency coupled with a low pressure drop so enabling an optimal efficiency/breathability balance. The so‐prepared filtering media were boosted with AgNPs to add a further protection against airborne microorganisms.

The filtration efficiency was tested at two different filtration velocities, as a function of different diameters of NaCl aerosol particles, comparable to that of a virus size. As well the air pressure drop, related to breathability, was measured and overall performances compared with standard requirements for FFP1 and FFP2 masks, matching the recommended limits (EN149‐2009 standard), even at the highest filtration velocities. The quality factor (QF) derived by considering both filtration efficiency and air pressure drop of 5‐layer samples was compared to commercial filter media, showing performance comparable with or better than the best commercial filter media.

Despite to PVA water solubility, the thermally treated electrospun patches showed a very good stability, even after 24 h of water contact, retaining their shape and porous structure. The presence of small quantity of silver in the electrospun matrix (around 30 mg m^−2^) showed excellent biocidal action against both Gram‐positive and Gram‐negative bacteria. In fact, nanofibers enriched of silver NPs showed percentage bacteria reductions of 99.6% against *S. aureus* and 100% against *E. coli*.

In summary, the optimal filtering efficiency towards nano‐aerosolized particles, combined with the low air drop‐pressure and the excellent antibacterial properties made the proposed PVA/Ag electrospun NFs optimum candidates for potential replaceable filtering media, to be incorporate in personal protective equipment facemasks or into indoor air purification devices.

## References

[1] Y. Wang, X. Zhao, X. Jiao, D. Chen, in Filtering Media by Electrospinning (Eds: M. L. Focarete, C. Gualandi, S. Ramakrishna), Springer International Publishing, Switzerland 2018, p. 47.

[2] F. Dotti, A. Varesano, A. Montarsolo, A. Aluigi, C. Tonin, G. Mazzuchetti, J. Ind. Text. 2007, 37, 151.

[3] H. El‐Sayed, C. Vineis, A. Varesano, S. Mowafi, R. A. Carletto, C. Tonetti, M. Abou Taleb, Nanotechnol. Rev. 2019, 8, 236.

[4] L. Li, M. W. Frey, T. B. Green, J. Eng. Fibers Fabr. 2006, 1, 1.

[5] A. Varesano, F. Rombaldoni, G. Mazzuchetti, C. Tonin, R. Comotto, Polym. Int. 2010, 59, 1606.

[6] A. Varesano, R. A. Carletto, G. Mazzuchetti, J. Mater. Process. Technol. 2009, 209, 5178.

[7] V. Guarino, A. Varesano, in Filtering Media by Electrospinning (Eds: M. L. Focarete, C. Gualandi, S. Ramakrishna), Springer International Publishing, Switzerland 2018, p. 1.

[8] F. Cengiz, T. A. Dao, O. Jirsak, Polym. Eng. Sci. 2010, 50, 936.

[9] A. Koski, K. Yim, S. Shivkumar, ACS Mater. Lett. 2004, 58, 493.

[10] C. Zhang, X. Yuan, L. Wu, Y. Han, J. Sheng, Eur. Polym. J. 2005, 41, 423.

[11] J. Wang, S. C. Kim, D. Y. H. Pui, Aerosol Sci. Technol. 2008, 39, 323.

[12] A. Figoli, C. Ursino, D. O. Sanchez Ramirez, R. A. Carletto, C. Tonetti, A. Varesano, M. P. De Santo, A. Cassano, C. Vineis, Polym. Eng. Sci. 2019, 59, 1472.

[13] N. Vitchuli, Q. Shi, J. Nowak, M. McCord, M. Bourham, X. Zhan, J. Appl. Polym. Sci. 2010, 116, 2181.

[14] W. C. Hinds, Aerosol Technology: Properties, Behavior and Measurement of Airborne Particles, 2nd ed., Wiley‐Interscience Publication, New York 1999, p. 182.

[15] F. Rombaldoni, K. Mahmood, A. Varesano, M. Bianchetto Songia, A. Aluigi, C. Vineis, G. Mazzuchetti, Surf. Coat. Technol. 2013, 216, 178.

[16] P. Heikkilä, A. Sipilä, M. Peltola, A. Harlin, A. Taipale, Text. Res. J. 2007, 77, 864.

[17] S. Zhang, W. S. Shim, J. Kim, Mater. Des. 2009, 30, 3659.

[18] D. Aussawasathien, C. Teerawattananon, A. Vongachariy, J. Membr. Sci. 2008, 315, 11.

[19] R. M. Hodge, G. M. Edward, G. P. Simon, Polymer 1996, 37, 1371.

[20] K. H. Hong, J. L. Park, I. H. Sul, J. H. Youk, T. J. Kang, J. Polym. Sci., Part B: Polym. Phys. 2006, 44, 2468.

[21] M. Lombardi, P. Palmero, M. Sangermano, A. Varesano, Polym. Int. 2011, 60, 234.

[22] S. Aryal, C. K. Kim, K. W. Kim, M. S. Khil, H. Y. Kim, Mater. Sci. Eng. C 2008, 28, 75.

[23] Y. Wu, W. Jia, Q. An, Y. Liu, J. Chen, G. Li, Nanotechnology 2009, 20, 245101. 19468171 10.1088/0957-4484/20/24/245101

[24] A. Cochis, S. Ferraris, R. Sorrentino, B. Azzimonti, C. Novara, F. Geobaldo, F. Truffa Giachet, C. Vineis, A. Varesano, A. S. Abdelgeliel, S. Spriano, L. Rimondini, J. Mater. Chem. B 2017, 5, 8366. 32264505 10.1039/c7tb01965c

[25] M. He, M. Chen, Y. Dou, J. Ding, H. Yue, G. Yin, X. Chen, Y. Cui, Polymer 2020, 12, 305. 10.3390/polym12020305 PMC7077473 32028586

[26] S. Tomaselli, D. O. Sanchez Ramirez, R. A. Carletto, A. Varesano, C. Vineis, S. Zanzoni, H. Molinari, L. Ragona, Macromol. Biosci. 2017, 17, 1600300. 10.1002/mabi.201600300 27805768

[27] K. Desai, K. Kit, J. Li, P. M. Davidson, S. Zivanovic, H. Meyer, Polymer 2009, 50, 3661.

[28] A. Varesano, C. Vineis, C. Tonetti, D. O. Sánchez Ramírez, G. Mazzuchetti, S. Ortelli, M. Blosi, A. L. Costa, Curr. Nanosci. 2015, 11, 41.

[29] G. U. Preethi, B. S. Unnikrishnan, M. M. Joseph, R. Shiji, T. T. Sreelekha, Curr. Sci. 2019, 116, 1735.

[30] A. Haider, I. K. Kang, Adv. Mater. Sci. Eng. 2015, 2015, 165257.

[31] Z. M. Xiu, J. Ma, P. J. J. Alvarez, Environ. Sci. Technol. 2011, 45, 9003. 21950450 10.1021/es201918f

[32] Y. J. Lee, W. S. Lyoo, J. Appl. Polym. Sci. 2010, 115, 2883.

[33] H. Maleki, S. Mathur, A. Klein, Polym. Eng. Sci. 2020, 60, 1221.

[34] A. L. Costa, M. Blosi, Patent WO 2016125070‐A1.

[35] V. Marassi, L. Di Cristo, S. G. J. Smith, S. Ortelli, M. Blosi, A. L. Costa, P. Reschiglian, Y. Volkov, A. Prina‐Mello, R. Soc. Open Sci. 2018, 5, 171113. 29410826 10.1098/rsos.171113 PMC5792903

[36] A. Podgorski, A. Bałazy, L. Gradon, Chem. Eng. Sci. 2006, 61, 6804.

